# Amphibians of Santa Teresa, Brazil: the hotspot further evaluated

**DOI:** 10.3897/zookeys.857.30302

**Published:** 2019-06-25

**Authors:** Rodrigo Barbosa Ferreira, Alexander Tamanini Mônico, Emanuel Teixeira da Silva, Fernanda Cristina Ferreira Lirio, Cássio Zocca, Marcio Marques Mageski, João Filipe Riva Tonini, Karen H. Beard, Charles Duca, Thiago Silva-Soares

**Affiliations:** 1 Programa de Pós-Graduação em Ecologia de Ecossistemas, Universidade Vila Velha, Campus Boa Vista, 29102-920, Vila Velha, ES, Brazil; 2 Department of Wildland Resources and the Ecology Center, Utah State University, Logan, UT, USA; 3 Instituto Nacional da Mata Atlântica/Museu de Biologia Prof. Mello Leitão, 29650-000, Santa Teresa, ES, Brazil; 4 Laboratório de Herpetologia, Departamento de Zoologia, Instituto de Ciências Biológicas, Universidade Federal de Minas Gerais, Avenida Antônio Carlos, 6627, Pampulha, Belo Horizonte, MG, Brazil; 5 Centro de Estudos em Biologia, Centro Universitário de Caratinga, Avenida Niterói, s/n, Bairro Nossa Senhora das Graças, 35300-000, Caratinga, MG, Brazil; 6 Department of Organismic and Evolutionary Biology, Harvard University, 26 Oxford St, Cambridge, MA, USA; 7 Museum of Comparative Zoology, Harvard University, 26 Oxford St, Cambridge, MA, USA

**Keywords:** Anura, Atlantic Forest, Caecilians, Diversity, Espírito Santo, Inventory

## Abstract

A checklist of the amphibians of Santa Teresa municipality, in southeastern Brazil is presented based on fieldwork, examination of specimens in collections, and a literature review. This new amphibian list of Santa Teresa includes 108 species, of which 106 (~98%) belong to Anura and two (~2%) to Gymnophiona. Hylidae was the most represented family with 47 species (43%). Compared to the previous amphibian lists for Santa Teresa, 14 species were added, 17 previously reported species were removed, and 13 species were re-identified based on recent taxonomic rearrangements. Of the 14 species added, 11 (79%) were first recorded during our fieldwork and specimen examination. It is also the first list of caecilians for Santa Teresa. This list suggests that Santa Teresa has 0.16 species per km^2^ (i.e., 108 species/683 km^2^), one of the highest densities of amphibian species in the world at a regional scale. This richness represents 78% of the 136 anurans from Espírito Santo state and 10% of the 1,080 amphibians from Brazil. We highlight the need for long-term monitoring to understand population trends and develop effective conservation plans to safeguard this remarkable amphibian richness.

## Introduction

Species checklists provide a scientific value to areas by identifying the richness that is threatened given anthropogenic actions. The Brazilian Amphibian Conservation Action plan recognizes that species lists are a scientific priority for many areas across Brazil (Verdade et al. 2012). For instance, Brazil’s Atlantic Forest is one of the most threatened global biodiversity hotspots and remains under-sampled given the high number of new species recently described (Lourenço-de-Moraes et al. 2014, Ferreira et al. 2015, Marciano-Jr et al. 2017). The Atlantic Forest has currently 12% of its historical range, which has resulted in the replacement of continuous forest to small remnants surrounded by human settlements, pastures, plantations, and roads (Ribeiro et al. 2009, Tabarelli et al. 2010). Thus, compiling data regarding the biodiversity of this tropical forest is a conservation priority, especially because several studies have detected changes and declines of some species (Heyer et al. 1988, Weygoldt 1989, Carvalho et al. 2017).

The Atlantic Forest harbors 625 anuran species and 14 caecilians (Rossa-Feres et al. 2017). The state of Espírito Santo, southeastern Brazil harbors 136 (22%) species listed for Atlantic Forest. The state’s most sampled area is the municipality of Santa Teresa, which comprises high functional and phylogenetic diversity of amphibians (Almeida et al. 2011, Campos et al. 2017, Lourenço-de-Moraes et al. 2019). There are conflicting reports regarding the species composition and richness in this area. The first species list for Santa Teresa recorded 102 anuran species (Rödder et al. 2007). However, the state list of anurans mentioned 92 species for Santa Teresa (Almeida et al. 2011). In recent years, new species have been described for Santa Teresa (e.g., Lourenço-de-Moraes et al. 2014, Ferreira et al. 2015, Taucce et al. 2018), some species have been reported for the first time in the area (Simon and Peres 2012), and there have been many taxonomic changes (e.g., Pimenta et al. 2014, Walker et al. 2016), indicating the need to update the species list of this anuran diversity hotspot.

Santa Teresa is also a hotspot for several other taxa, such as plants (Thomaz and Monteiro 1997), birds (Simon 2000), butterflies (Brown and Freitas 2000), and small mammals (Passamani et al. 2000). Due to its remarkable biological importance, it is essential to keep the species lists updated. Here, we present an updated species list of the amphibians for Santa Teresa based on many years of fieldwork, examination of specimens from scientific collections, and literature review.

## Materials and methods

### Study area

**Figure 1.**
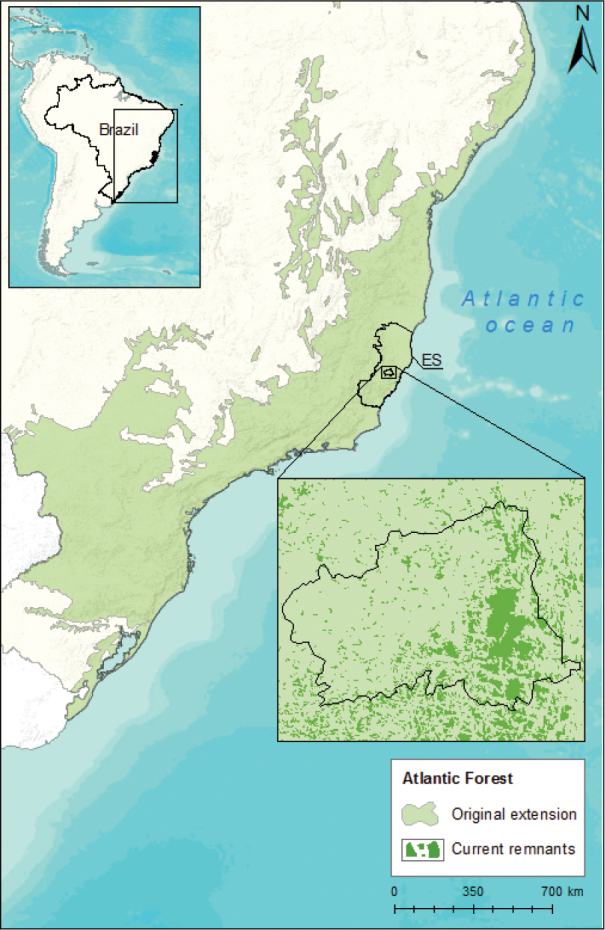
Location of the municipality of Santa Teresa, southeastern Brazil. Forest remnants from SOS Mata Atlântica (2014).

The municipality of Santa Teresa has 683 km^2^ and is located in the mountainous region (altitude range: ~120–1099 m a.s.l.) of Espírito Santo state, southeastern Brazil (19°56'14"S, 40°35'52"W; Figure 1). Santa Teresa encompasses the southern portion of Bahia Coastal Forests ecoregion, and northern portion of Serra do Mar ecoregion in the Atlantic Forest (Olson et al. 2001, Scaramuzza et al. 2011, Campos and Lourenço-de-Moraes 2017, Silva et al. 2018).

The predominant vegetation types are montane and sub-montane rainforests (Rizzini 1979), characterized by non-deciduous trees with lead buds without protection against drought (Brasil 1983). Santa Teresa was mostly forested until the arrival of European settlers in 1874. Currently, the municipality has 42% of its original forest cover inside and surrounding three protected areas: the Reserva Biológica Augusto Ruschi (3,598 ha), the Estação Biológica de Santa Lúcia (440 ha), and the Parque Natural de São Lourenço (22 ha) (SOS Mata Atlântica and Inpe 2013). Outside these protected areas, forest remnants are in private properties and mostly restricted to hilltops while the valleys are dominated by different types of human-modified matrix (e.g., coffee plantations, *Eucalyptus* spp. plantations, abandoned pastures, and settlements; Ferreira et al. 2016).

The climate of Santa Teresa is classified as oceanic climate without dry season and with temperate summer (Cfb) according to Köppen classification (Alvares et al. 2013). Mean annual precipitation is 1,868 mm with highest rainfall in November and lowest in June, when the mean rainfall is less than 60 mm (Mendes and Padovan 2000). Mean annual temperature is 20 °C (range: 14.3–26.2 °C, Thomaz and Monteiro 1997).

### Data sampling

The species list presented in this study has been compiled in part using field surveys conducted by the authors from 2006 to 2019, and also through the evaluation of specimens in zoological collections (see Appendix I) and a literature review.

During field surveys, we conducted intensive sampling across Santa Teresa using audio and visual searches inside bromeliads, in the leaf litter, and in water bodies (see Dodd 2010). We released easily identified and extensively vouchered (> 30 specimens) species but took those species with more complex identification back to laboratory. To do this, we kept amphibians in moist plastic tubes or plastic bags to prevent dehydration. Some specimens were euthanized by ventral application of 7.5% to 20% benzocaine, preserved using 10% formalin and then transferred to 70% ethanol (American and Veterinary Medical Association 2013, CEBEA/CFMV 2013).

We also reviewed the literature and compiled records of amphibians for Santa Teresa. In addition, we examined specimens deposited in the following institutions: Coleção de Anfíbios Célio F. B. Haddad (CFBH), Universidade Estadual Paulista (UNESP); Museu de Biologia Mello Leitão (MBML), Instituto Nacional da Mata Atlântica (INMA); Universidade Federal de Minas Gerais (UFMG); Museu Nacional, Universidade Federal do Rio de Janeiro (MNRJ); Museu de Zoologia Prof. Adão José Cardoso (ZUEC), Universidade Estadual de Campinas (UNICAMP); Museu de Zoologia, Universidade de São Paulo (MZUSP); and Smithsonian National Museum of Natural History (USNM) (see Appendix I). We followed Frost (2019) for taxonomic arrangements.

## Results

**Figure 2.**
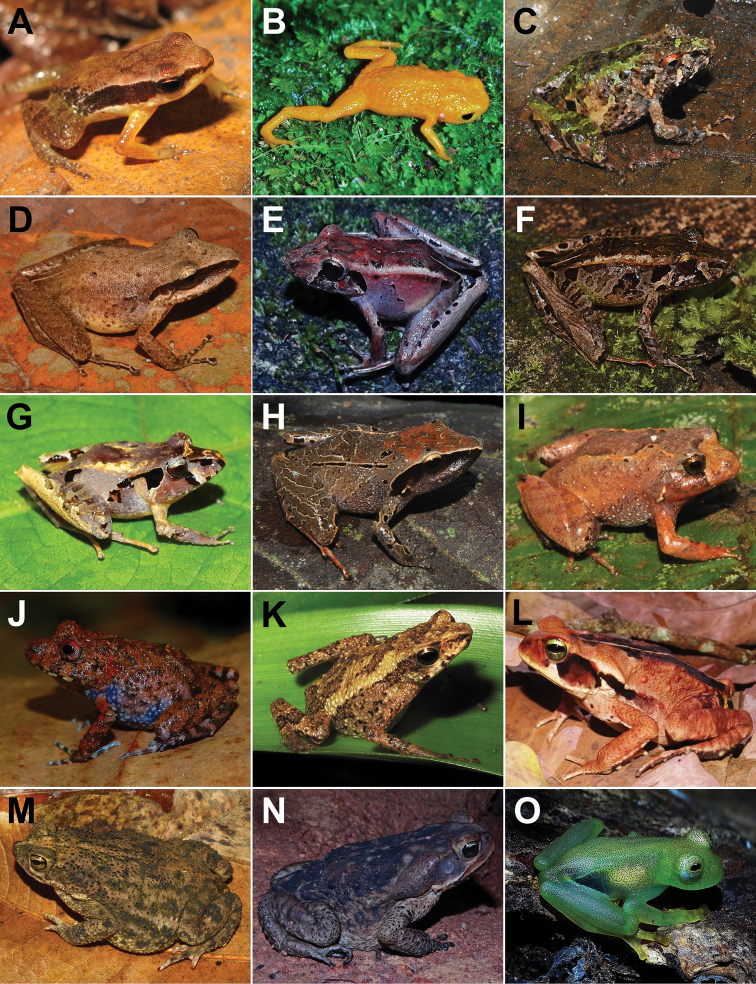
Amphibians from Santa Teresa: **A***Allobatescapixaba***B***Brachycephalusalipioi***C***Ischnocnemaabdita***D***Ischnocnemacolibri***E**Ischnocnemacf.nasuta**F**Ischnocnemaaff.guentheri**G***Ischnocnemaoea***H**Ischnocnemagr.parva sp. new 1 **I**Ischnocnemagr.parva sp. new 2 **J***Ischnocnemaverrucosa***K***Dendrophryniscuscarvalhoi***L***Rhinellacrucifer***M***Rhinellagranulosa***N***Rhinelladiptycha***O**Vitreoranaaff.eurygnatha. Photographs by JFR Tonini (**A**), CN Fraga (**B**), RB Ferreira (**C, D, H, I, K**), AT Mônico (**E, G, J, K, L, M, N, O**), T Silva-Soares (**F**).

**Figure 3.**
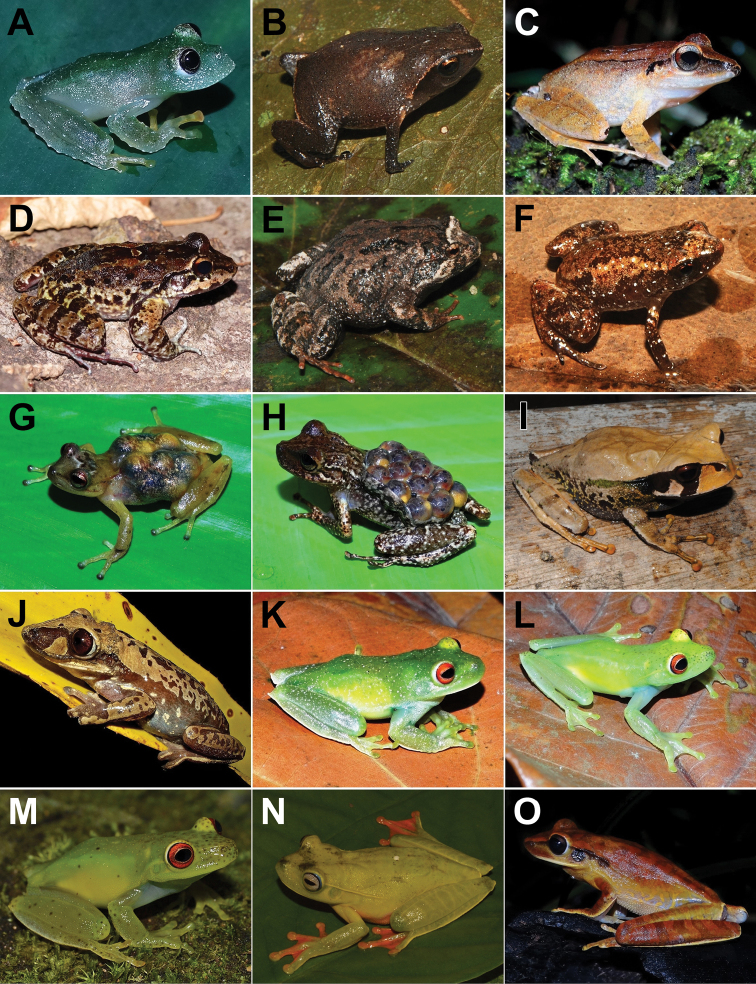
Amphibians from Santa Teresa: **A***Vitreoranauranoscopa***B***Euparkerellatridactyla***C***Haddadusbinotatus***D***Thoropamiliaris***E***Zachaenuscarvalhoi***F***Adelophryneglandulata***G**Fritzianaaff.fissilis**H***Fritzianatonimi***I***Gastrothecamegacephala***J***Aparasphenodonbrunoi***K***Aplastodiscuscavicola***L**Aplastodiscusaff.eugenioi**M***Aplastodiscusweygoldti***N***Boanaalbomarginata***O***Boanaalbopunctata*. Photographs by AT Mônico (**A, C, D, G, H, K, L, O**), RB Ferreira (**B, E, F, I, N**), C Zocca (**J**), T Silva-Soares (**M**).

**Figure 4.**
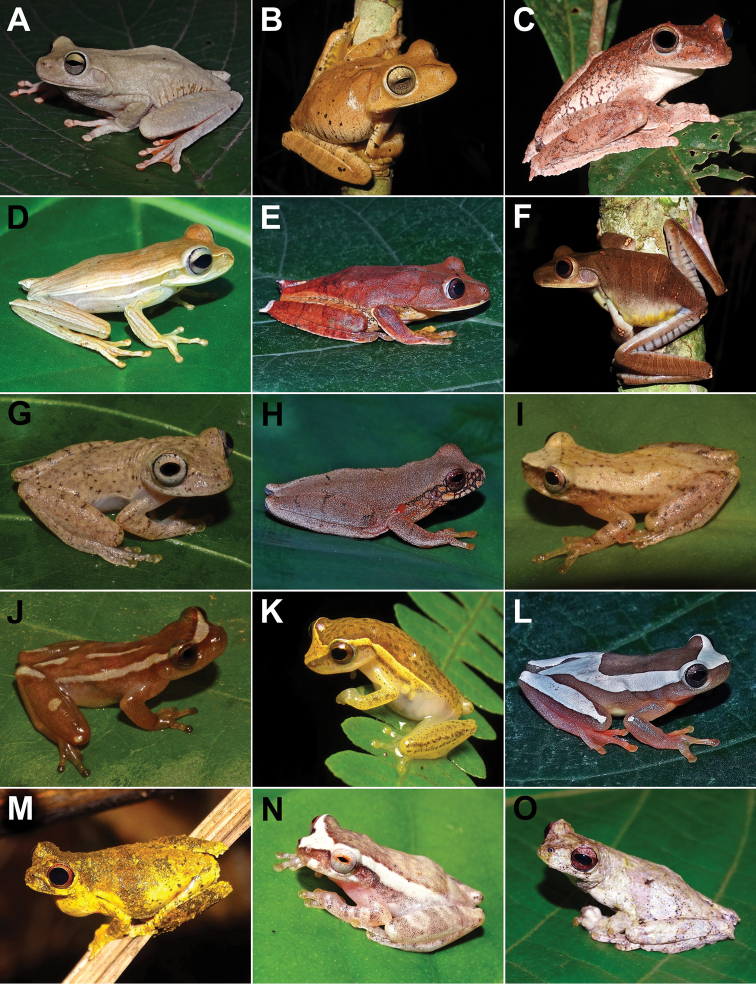
Amphibians from Santa Teresa: **A***Boanacrepitans***B***Boanafaber***C***Boanapardalis***D***Boanapolytaenia***E***Boanasemilineata***F***Bokermannohylacaramaschii***G***Dendropsophusberthalutzae***H***Dendropsophusbipunctatus***I***Dendropsophusbranneri***J***Dendropsophusbromeliaceus***K***Dendropsophusdecipiens***L***Dendropsophuselegans***M***Dendropsophusgiesleri***N***Dendropsophushaddadi***O***Dendropsophusmicrops*. Photographs by AT Mônico (**A, C, D, E, F, H, L, M, N, O**), RB Ferreira (**B, G, I, J**), ET Silva (**K**).

**Figure 5.**
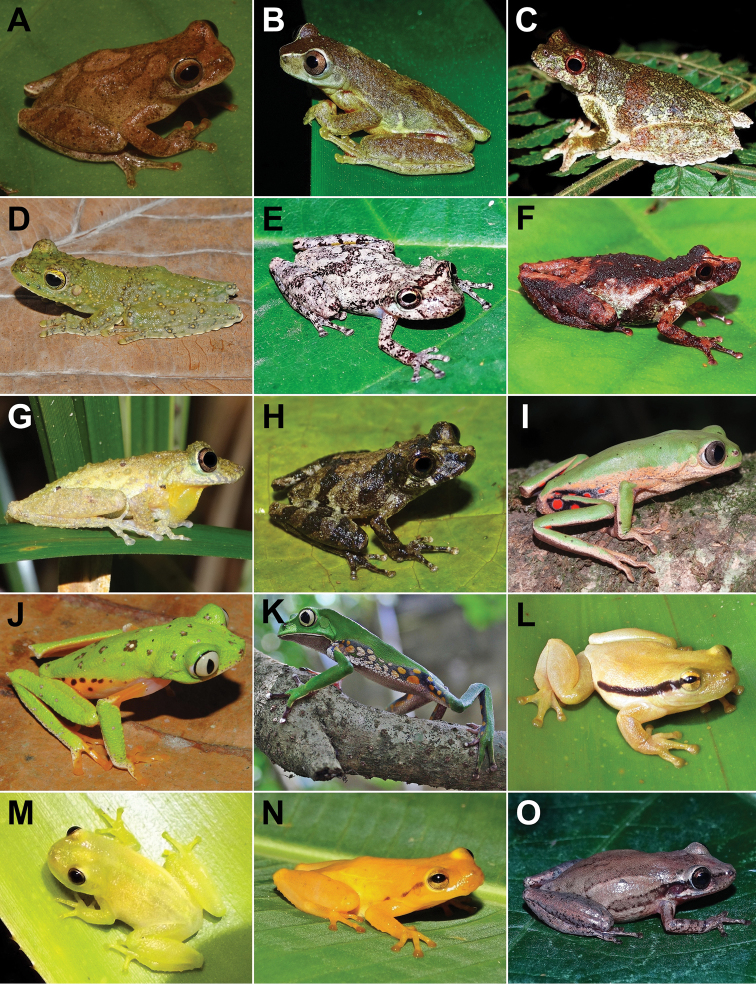
Amphibians from Santa Teresa: **A***Dendropsophusminutus***B***Dendropsophusruschii***C***Dendropsophusseniculus***D***Itapotihylalangsdorffii***E***Ololygonarduous***F***Ololygonargyreornata***G***Ololygonheyeri***H***Ololygonkautskyi***I**Pithecopusaff.rohdei**J***Phasmahylaexilis***K***Phyllomedusaburmeisteri***L***Phyllodyteskautskyi***M***Phyllodytesluteolus***N**Phyllodytesaff.luteolus**O***Scinaxalter*. Photographs by RB Ferreira (**A, D, J**), AT Mônico (**B, C, E, F, G, I, K, N, O**), T Silva-Soares (**H**), CZ Zocca (**L, M**).

**Figure 6.**
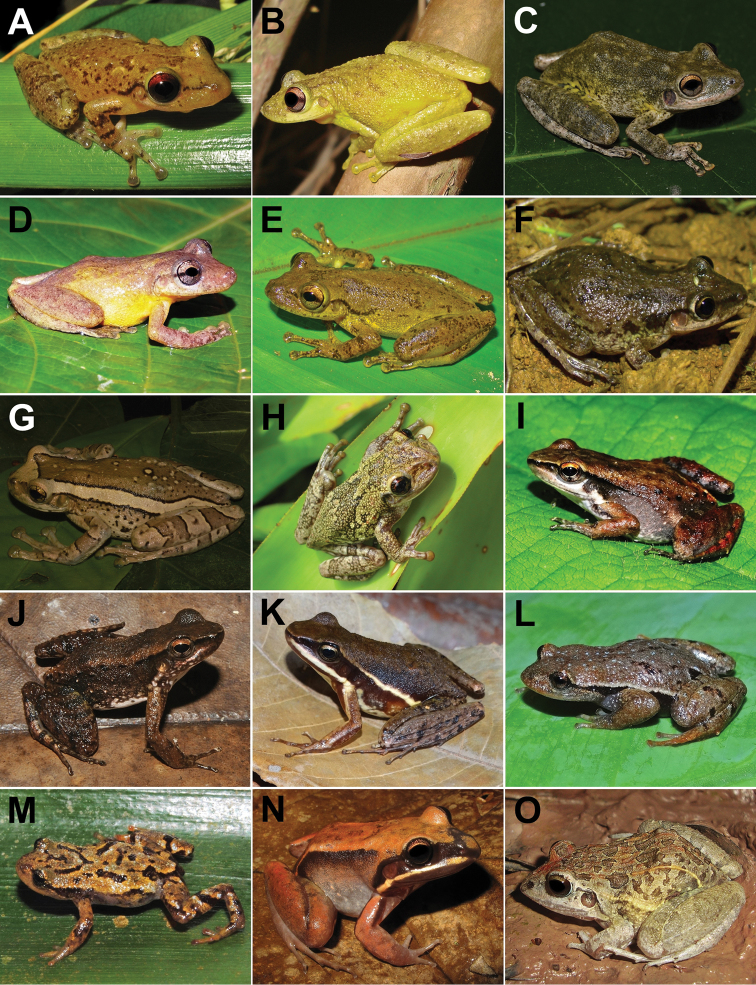
Amphibians from Santa Teresa: **A***Scinaxcuspidatus***B***Scinaxeurydice***C***Scinaxfuscovarius***D***Scinaxhayii***E**Scinaxaff.perereca**F**Scinaxcf.x-signatus**G***Trachycephalusmesophaeus***H***Trachycephalusnigromaculatus***I**Crossodactylusaff.gaudichaudii**J***Crossodactylustimbuhy***K***Hylodeslateristrigatus***L***Crossodactylodesbokermanni***M***Crossodactylodesizecksohni***N***Leptodactyluscupreus***O***Leptodactylusfuscus*. Photographs by ET Silva (**A, E, F**), CZ Zocca (**B, O**), T Silva-Soares (**C**), AT Mônico (**D, I, K, L**), RB Ferreira (**G, H, J, M**), JFR Tonini (**N**).

**Figure 7.**
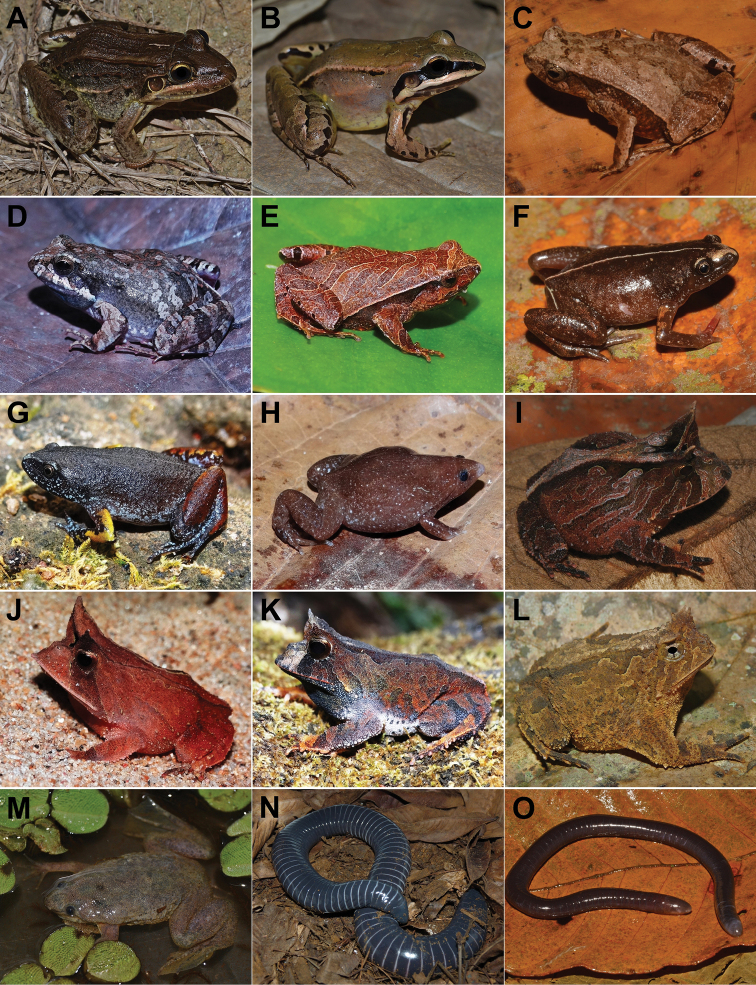
Amphibians from Santa Teresa: **A**Leptodactylusaff.latrans**B**Leptodactylusaff.spixi**C***Physalaemuscrombiei***D***Physalaemuscuvieri***E***Physalaemusmaculiventris***F***Chiasmocleiscapixaba***G***Chiasmocleisschubarti***H***Myersiellamicrops***I***Proceratophrysboiei***J***Proceratophryslaticeps***K***Proceratophryspaviotii***L***Proceratophrysschirchi***M**Pipaaff.carvalhoi**N***Siphonopsannulatus***O***Siphonopshardyi*. Photographs by T Silva-Soares (**A, B, L, M, N**), AT Mônico (**C, D, E, G, H, J, K**), RB Ferreira (**F, I, O**).

We recorded 108 amphibian species for Santa Teresa, of which 106 (98%) belong to Anura (16 families and 41 genera) and two (2%) to Gymnophiona (one family and one genus) (Table 1; Figure 2, 3, 4, 5, 6, 7). The most represented families were Hylidae with 47 species (43%), Brachycephalidae with 11 species (10%), and Leptodactylidae with 10 species (9%). Santa Teresa is currently the type locality for 23 species (20%) (Table 1). So far, four species (3%) are only found in Santa Teresa such as *Crossodactylodesizecksohni*, *Crossodactylustimbuhy*, *Ischnocnemacolibri* and *Ischnocnemaepipeda*. The species density of Santa Teresa is 0.16 species per km^2^ (i.e., 108 species/683 km^2^).

Compared to previous anuran lists for Santa Teresa, we added 14 species, removed 17 previously reported species, and re-determined 14 species based on recent taxonomic rearrangements. Out of the 14 added species, 11 (79%) were first recorded during our fieldwork and specimen examination, two (14%) records were from the literature, and one (7%) new record was from pers. comm. (*Gastrothecaernestoi*; MT Rodrigues, field number MTR 34695).

Fourteen species classified to morphotypes are new species, such as Aplastodiscusaff.eugenioi (M Mongin, pers. comm.), Brachycephalusaff.didactylus (TSS, in. prep.), Crossodactylusaff.gaudichaudii (R Montesinos, in. prep.), Fritzianaaff.fissilis (RBF, pers. obs.), Ischnocnemaaff.parva sp. 1 (CAG Cruz, in. prep.), Ischnocnemaaff.parva sp. 2 (TSS, in. prep.), Leptodactylusaff.spixi (L Nascimento, in. prep.), Ololygonaff.heyeri (J Lacerda, pers. comm.), Phyllodytesaff.luteolus (ATM, in. prep.), Pipaaff.carvalhoi (PV Scherrer, in. prep.), Pithecopusaff.rohdei (D Baêta, pers. comm.), Scinaxaff.perereca (TSS, pers. comm.), Thoropaaff.lutzi (CL Assis, pers. comm.), and Vitreoranaaff.eurygnatha (R Pontes, in. prep.).

**Table 1.** Amphibian species of Santa Teresa municipality, Espírito Santo state, Southeastern Brazil. An asterisk * indicates a taxonomic change.

Species by Family	Type locality	Our study	Almeida et al. 2011	Rödder et al. 2007
** AROMOBATIDAE **				
*Allobatescapixaba* (Lutz, 1925)		X	X	X*
** BRACHYCEPHALIDAE **				
*Brachycephalusalipioi* Pombal & Gasparini, 2006		X	X	–
Brachycephalus aff. didactylus		X	–	–
*Ischnocnemaabdita* Canedo & Pimenta, 2010	X	X	X	–
*Ischnocnemacolibri* Taucce, Canedo, Parreiras, Drummond, Nogueira-Costa & Haddad, 2018	X	X	–	–
*Ischnocnemaepipeda* (Heyer, 1984)	X	X	X	X
Ischnocnema aff. guentheri		X	X*	X*
Ischnocnemacf.nasuta (Lutz, 1925)		X	X*	X*
*Ischnocnemaoea* (Heyer, 1984)	X	X	X	X
Ischnocnemaaff.parva sp. 1		X	X*	X*
Ischnocnemaaff.parva sp. 2		X	–	–
*Ischnocnemaverrucosa* Reinhardt & Lütken, 1862		X	X	X
** BUFONIDAE **				
*Dendrophryniscuscarvalhoi* Izecksohn, 1994	X	X	X	X
*Rhinellacrucifer* (Wied-Neuwied, 1821)		X	X	X
*Rhinellagranulosa* (Spix, 1824)		X	X	X
*Rhinelladiptycha* (Cope, 1862)		X	X	X
** CENTROLENIDAE **				
Vitreorana aff. eurygnatha		X	X*	X*
*Vitreoranauranoscopa* (Müller, 1924)		X	X	X
** CERATOPHRYIDAE **				
*Ceratophrysaurita* (Raddi, 1823)		X	X	X*
** CRAUGASTORIDAE **				
*Euparkerellatridactyla* Izecksohn, 1988	X	X	X	X
*Haddadusbinotatus* (Spix, 1824)		X	X	X
** CYCLORAMPHIDAE **				
*Cycloramphusfuliginosus* Tschudi, 1838		X	X	X
Thoropa aff. lutzi		X	X*	–
*Thoropamiliaris* (Spix, 1824)		X	X	X
*Thoropapetropolitana* (Wandolleck, 1907)		X	X	–
*Zachaenuscarvalhoi* Izecksohn, 1983	X	X	X	X
** ELEUTHERODACTYLIDAE **				
*Adelophryneglandulata* Lourenço-de-Moraes, Ferreira, Fouquet & Bastos, 2014	X	X	X*	–
** HEMIPHRACTIDAE **				
Fritziana aff. fissilis		X	X*	X*
*Fritzianatonimi* Walker, Gasparini, Haddad, 2016	X	X	X*	X*
*Gastrothecaalbolineata* (Lutz & Lutz, 1939)		X	X	–
*Gastrothecaernestoi* Miranda-Ribeiro, 1920		X	–	–
*Gastrothecamegacephala* Izecksohn, Carvalho-e-Silva & Peixoto, 2009		X	–	–
** HYLIDAE **				
*Aparasphenodonbrunoi* Miranda-Ribeiro, 1920		X	–	X
*Aplastodiscuscavicola* (Cruz & Peixoto, 1985)	X	X	X	X
Aplastodiscus aff. eugenioi		X	–	–
*Aplastodiscusweygoldti* (Cruz & Peixoto, 1987)	X	X	X	X
*Boanaalbomarginata* (Spix, 1824)		X	X	X
*Boanaalbopunctata* (Spix, 1824)		X	X	X
*Boanacrepitans* (Wied-Neuwied, 1824)		X	X	X
*Boanafaber* (Wied-Neuwied, 1821)		X	X	X
*Boanapardalis* (Spix, 1824)		X	X	X
*Boanapolytaenia* (Cope, 1870)		X	X	–
*Boanasemilineata* (Spix, 1824)		X	X	X
*Bokermannohylacaramaschii* (Napoli, 2005)	X	X	X	X
*Dendropsophusberthalutzae* (Bokermann, 1962)		X	X	X
*Dendropsophusbipunctatus* (Spix, 1824)		X	X	X
*Dendropsophusbranneri* (Cochran, 1948)		X	X	X
*Dendropsophusbromeliaceus* Ferreira, Faivovich, Beard & Pombal, 2015	X	X	–	–
*Dendropsophusdecipiens* (Lutz, 1925)		X	X	X
*Dendropsophuselegans* (Wied-Neuwied, 1824)		X	X	X
*Dendropsophusgiesleri* (Mertens, 1950)		X	X	X
*Dendropsophushaddadi* (Bastos & Pombal, 1996)		X	X	X
*Dendropsophusmicrops* (Peters, 1872)		X	X	X
*Dendropsophusminutus* (Peters, 1872)		X	X	X
*Dendropsophusruschii* (Weygoldt & Peixoto, 1987)		X	X	X
*Dendropsophusseniculus* (Cope, 1868)		X	X	X
*Itapotihylalangsdorffii* (Duméril & Bibron, 1841)		X	X	X
*Ololygonarduous* (Peixoto, 2002)	X	X	X	X
*Ololygonargyreornata* (Miranda-Ribeiro, 1926)		X	X	X
Ololygoncf.flavoguttata (Lutz & Lutz, 1939)		X	–	–
Ololygon aff. heyeri		X	–	–
*Ololygonheyeri* Peixoto & Weygoldt, 1986	X	X	X	X
*Ololygonkautskyi* Carvalho-e-Silva & Peixoto, 1991		X	X	X
*Phasmahylaexilis* (Cruz, 1980)	X	X	X	X
*Phrynomedusamarginata* (Izecksohn & Cruz, 1976)	X	X	X	X
*Phyllodyteskautskyi* Peixoto & Cruz, 1988		X	–	–
*Phyllodytesluteolus* (Wied-Neuwied, 1824)		X	X	X
Phyllodytes aff. luteolus		X	–	–
*Phyllomedusaburmeisteri* Boulenger, 1882		X	X	X
Pithecopus aff. rohdei		X	X*	X*
*Scinaxalter* (Lutz, 1973)		X	X	X
*Scinaxcuspidatus* (Lutz, 1925)		X	X	X
*Scinaxeurydice* (Bokermann, 1968)		X	X	X*
*Scinaxfuscovarius* (Lutz, 1925)		X	X	X
*Scinaxhayii* (Barbour, 1909)		X	X	X*
Scinax aff. perereca		X	–	–
Scinaxcf.x-signatus (Spix, 1824)		X	X	X
*Trachycephalusmesophaeus* (Hensel, 1867)		X	X	X
*Trachycephalusnigromaculatus* Tschudi, 1838		X	X	X
** HYLODIDAE **				
Crossodactylus aff. gaudichaudii		X	X	X*
*Crossodactylustimbuhy* Pimenta, Cruz & Caramaschi, 2014	X	X	X*	X*
Hylodescf.babax Heyer, 1982		X	X*	X*
*Hylodeslateristrigatus* (Baumann, 1912)		X	X	X
*Megaelosiaapuana* Pombal, Prado & Canedo, 2003		X	X	X*
** LEPTODACTYLIDAE **				
*Crossodactylodesbokermanni* Peixoto, 1983	X	X	X	X
*Crossodactylodesizecksohni* Peixoto, 1983	X	X	X	X
*Leptodactyluscupreus* Caramaschi, Feio & São Pedro, 2008		X	X	–
*Leptodactylusfuscus* (Schneider, 1799)		X	X	X
Leptodactylusaff.latrans (Steffen, 1815)		X	X*	X*
Leptodactylus aff. spixi		X	X*	X*
*Physalaemuscrombiei* Heyer & Wolf, 1989	X	X	X	X
*Physalaemuscuvieri* Fitzinger, 1826		X	X	X
*Physalaemusmaculiventris* (Lutz, 1925)		X	X	–
Physalaemuscf.olfersii (Lichtenstein & Martens, 1856)		X	X*	X*
** MICROHYLIDAE **				
*Chiasmocleiscapixaba* Cruz, Caramaschi & Izecksohn, 1997		X	–	–
*Chiasmocleisschubarti* Bokermann, 1952		X	–	X
*Myersiellamicrops* (Duméril & Bibron, 1841)		X	X	X
** ODONTOPHRYNIDAE **				
*Macrogenioglottusalipioi* Carvalho, 1946		X	X	X
*Proceratophrysboiei* (Wied-Neuwied, 1824)		X	X	X
*Proceratophryslaticeps* Izecksohn & Peixoto, 1981		X	X	X
*Proceratophrysmoehringi* Weygoldt & Peixoto, 1985	X	X	X	X
*Proceratophryspaviotii* Cruz, Prado & Izecksohn, 2005	X	X	X	X
*Proceratophrysphyllostomus* Izecksohn, Cruz & Peixoto, 1999		X	X	X
*Proceratophrysschirchi* (Miranda-Ribeiro, 1937)		X	X	X
** PIPIDAE **				
Pipa aff. carvalhoi		X	X*	X*
** RANIDAE **				
*Lithobatescatesbeianus* (Shaw, 1802)		X	–	–
** SIPHONOPIDAE **				
*Siphonopsannulatus* (Mikan, 1822)		X	–	–
*Siphonopshardyi* Boulenger, 1888		X	–	–

## Discussion

The current number of 106 anuran species for Santa Teresa is remarkable, and represents 78% of the 136 species listed for Espírito Santo state (Almeida et al. 2011, Rossa-Feres et al. 2017), 10% of the 1,080 species listed for Brazil (Segalla et al. 2016), and 1.5% of the 7,068 species listed worldwide (AmphibiaWeb 2019). To date, the species density (i.e., 0.16 species per km^2^) is one of the highest in the world at regional scale. For instance, Yasuní National Park in Ecuador has 0.015 species per km^2^ (i.e., 150 species/9,820 km^2^; Bass et al. 2010); Tambopata in southern Peru has 0.06 species per km^2^ (i.e., 99 species/1,600 km^2^; Doan and Arriaga 2002); Iquitos region of northern Loreto in Peru has 0.012 species per km^2^ (i.e., 141 species/11,310 km^2^; IUCN 2008, Rodríguez and Duellman 1994); and Leticia in Colombia has 0.13 species per km^2^ (i.e., 123 species/927 km^2^; Lynch 2005). Several other localities across the Atlantic Forest also have remarkable amphibian richness at local scales. For example, Reserva Biológica de Paranapiacaba in Sao Paulo state has 20.5 species per km^2^ (69 species/3.36 km^2^; Verdade et al. 2009); Fazenda Vista Bela in Bahia state has 7.3 species per km^2^ (34 species/4.65 km^2^; Silvano and Pimenta 2003); and Reserva Particular do Patrimônio Natural Serra Bonita has 4 species per km^2^ (80 species/20 km^2^; Dias et al. 2014). We acknowledge that amphibian richness per area represents just a first approximation for practical spatial comparisons and that the lack of adequate surveys in more unexplored diverse regions (e.g., Indonesia, New Guinea, and the Congo Basin) may reveal remarkable amphibian richness. So far, Brazil’s Atlantic Forest and the northwest Amazon are considered the world’s greatest amphibian diversity on a landscape scale (Young et al. 2004, Bass et al. 2010).

The two species of Gymnophiona (*Siphonopsannulatus* and *S.hardyi*) were found during our fieldwork but have been reported previously for Santa Teresa (Caramaschi et al. 2004, Maciel et al. 2009). The former has a wide distribution in South America from Colombia to Argentina (Frost 2018). The latter has a more restricted distribution in southeastern of Brazil (Maciel et al. 2009, Frost 2018). Caecilians are difficult to sample due to the subterranean or aquatic habits (Oommen et al. 2000, Maciel and Hoogmoed 2011). Although amphibians are dramatically declining (Stuart et al. 2004), the conservation status of caecilians is largely unknown due to the lack of information on their biology, ecology and natural history (Wilkinson and Nussbaum 1999, Oommen et al. 2000, Gower and Wilkinson 2005). It is likely more species of caecilians will be recorded in Santa Teresa if the use of sampling methods specific for these taxa is applied in the field.

Our fieldwork since 2005 in Santa Teresa has made notable contributions toward the knowledge of local amphibians. It has resulted in the description of three new species for the municipality (i.e., *Adelophryneglandulata* in Lourenço-de-Moraes, Ferreira, Fouquet, Bastos 2014, *Dendropsophusbromeliaceus* in Ferreira, Faivovich, Beard, Pombal 2015, and *Ischnocnemacolibri* in Taucce, Canedo, Parreiras, Drummond, Nogueira-Costa, Haddad 2018). Furthermore, our fieldwork found individuals of 13 morphospecies that are currently under formal description (i.e., *Aplastodiscus* aff. *eugenioi, Brachycephalus*aff.didactylus, Crossodactylusaff.gaudichaudii, *Fritziana* aff. *fissilis, Ischnocnema*aff.parva sp. 1, Ischnocnemaaff.parva sp. 2, Leptodactylusaff.spixi, Ololygonaff.heyeri, Phyllodytesaff.luteolus, Pipaaff.carvalhoi, *Pithecopus* aff. *rohdei, Scinax*aff.perereca, and Vitreoranaaff.eurygnatha). The discovery of new species, morphospecies, and new records for Santa Teresa may be due to our sampling in remote forested areas and rocky outcrops through both visual bromeliad surveys and active leaf-litter searches (Ferreira et al. 2016).

Our species list resolved some differences between the previous species lists of Santa Teresa, which had disagreements on 11 species (e.g., Rödder et al. 2007, Almeida et al. 2011). We confirmed that *Chiasmocleisschubarti* occurs in Santa Teresa based on several individuals sampled in the Reserva Biológica Augusto Ruschi, whereas Almeida et al. (2011) challenged previous records of this species listed in Cruz et al. (1997) and Rödder et al. (2007). We also confirmed the presence of *Aparasphenodonbrunoi* and *Trachycephalusnigromaculatus* reported in Santa Teresa at the buffer zone of the Parque Municipal do Goiapaba-Açu (Ramos and Gasparini 2004). Almeida et al. (2011) challenged the record of *Rhinellahoogmoedi* referring to the species as Rhinellagr.margaritifer, because the former species was not mentioned in Rödder et al. (2007). We agree with Almeida et al. (2011) regarding the exclusion of several species from Rödder et al. (2007), such as Bokermannohylaaff.nanuzae (MBML 4528 corresponds to *B.caramaschii*), *Dendrophryniscus* sp. (MBML 3841 corresponds to *D.carvalhoi*), Ischnocnemacf.juipoca (MBML 5737 corresponds to *I.abdita*), *I.lactea* (MBML 1143 corresponds to *I.abdita*), *Physalaemusaguirrei* (MBML 2803-04 correspond to P.cf.olfersii), and *Proceratophrysappendiculata* (MBML 1154 corresponds to *P.schirchii*). Rödder et al. (2007) and Almeida et al. (2011) listed *Leptodactylusnatalensis* for Santa Teresa but the voucher specimens (MBML 3909-10) were misidentified and actually refer to individuals of L.aff.spixi. Rödder et al. (2007) listed Allobatescf.olfersioides following Verdade and Rodrigues (2007) who placed *A.capixaba* as synonym of *A.olfersioides*. Studies on *Allobates* indicate *A.capixaba* is a valid taxon (e.g., Bokermann 1967; Forti et al. 2017), which agrees with Almeida et al. (2011). Fieldwork should be conducted in the vicinities of Santa Teresa to confirm the presence of *Brachycephalusalipioi*. This species has not been found in Santa Teresa since 1952 when the municipality was larger than it is today (Pombal and Gasparini 2006).

The wide elevational range of Santa Teresa (~120–1099 m a.s.l.) partially explains the high richness of amphibian species. Species typical of both Atlantic Forest lowlands (e.g., *Allobatescapixaba*, *Chiasmocleisschubarti*, *C.capixaba*, *Dendropsophusbipunctatus*, *Ololygonargyreornata*) and highlands (e.g., *Aplastodiscuscavicola*, *Bokermannohylacaramaschii*, *Dendropsophusruschii*) occur in Santa Teresa, which suggest that the elevational gradient influences species composition. The high amphibian diversity also may be related to edaphic and topographic heterogeneity, which is known to cause speciation in many Atlantic Forest species occurring in mountainous areas (Carnaval et al. 2014). The high altitude and proximity to the Atlantic Ocean favors frequent orographic rain, which contribute to the meeting the reproductive requirements of amphibians. It is worth highlighting that Santa Teresa is one of the most sampled regions for amphibians in the Atlantic Forest (Rödder et al. 2007, Almeida et al. 2011, Zocca et al. 2014, Ferreira et al. 2016). About 3,800 anuran specimens collected in Santa Teresa were found housed in Brazilian collections (ET Silva, pers. obs.). This high sampling effort, which is comparable to only a few localities in the Atlantic Forest, may also account for such high species richness.

## Conservation remarks

Amphibians from Santa Teresa have faced several anthropogenic disturbances over the last couple of decades. The first report on amphibian declines for Santa Teresa was in 1989 (see Weygoldt 1989). During long-term sporadic samplings (i.e., 1975 and 1988), Weygoldt (1989) reported the decline and possible disappearances of eight species (updated taxonomy: *Allobatescapixaba*, Crossodactylusaff.gaudichaudii, *C.timbuhy*, *Cycloramphusfuliginosus*, *Hylodeslateristrigatus*, H.cf.babax, *Phasmahylaexilis*, and Vitreoranaaff.eurygnatha). To our knowledge, *Cycloramphusfuliginosus* and Hylodescf.babax have not been recorded after Weygoldt (1989). Additionally, *Thoropapetropolitana*, a frog not mentioned by Weygoldt (1989) has disappeared with no recent records along its entire range (Haddad et al. 2016). Several potential causes of these declines were mentioned by Weygoldt (1989), such as pollution (acid rain and pesticides), long-term climatic changes, and epidemic diseases. Weygoldt (1986) mentioned that Crossodactyluscf.dispar (currently *C.timbuhy*) was rare in Santa Teresa and later reported its decline. However, during our surveys we easily found this species on creeks across Santa Teresa. We cannot assess whether species declines are actually happening in Santa Teresa because only long-term and species-specific studies can precisely understand population trends.

Over the decades, we have noted population disappearances of anurans in Santa Teresa. The construction of condominiums and vacation ranches has intensified over the last decade and consequently increased deforestation of primary forest. We have also observed the expansion of the non-native *Eucalyptus* spp. plantations near primary and secondary forests and the replacement of coffee plantations. Another unmeasured concern is the increasing record of morphological anuran deformities, which is likely a result of pesticides used on crops (e.g., Mônico et al. 2016), including inside the buffer zone of the largest forest reserve (i.e., Reserva Biológica Augusto Ruschi; pers. obs.). The report of the invasive frog, *Lithobatescatesbeianus*, in Santa Teresa (see Ferreira and Lima 2012) should be further evaluated to monitor its establishment, and possible spread and impacts. We emphasize the need to sample the surroundings of the nearby breeding farms of *L.catesbeianus*. Studies have shown that non-native *L.catesbeianus* can be voracious predators of native anurans and vectors of diseases (Schloegel et al. 2010, Silva et al. 2011, Boelter et al. 2012).

The landscape configuration of Santa Teresa does not safeguard the maintenance of amphibian reproduction outside protected reserves because forests on private properties are mostly restricted to hilltops and non-natural matrix habitats occupy most valleys. Because water-body breeding species migrate toward reproductive habitats in the valleys, these species face severe threats, such as the risk of predation and desiccation (Becker et al. 2007, Ferreira et al. 2016). In addition, pollution of creeks and streams further strengthen conservation concern of lotic body breeders. We reinforce the need of studies focused on the threats amphibians are facing in the region to provide knowledge for conservationists and reserves managers to safeguard the local diversity.

Santa Teresa is an important hotspot for amphibian conservation due to its high richness and number of endemic species. The discovery of several new species further emphasizes the importance of this mountainous region for amphibian conservation. Even though Santa Teresa and its surrounding areas in southeastern Brazil are one of the most sampled regions in the Atlantic Forest, the region still harbors numerous remote areas that have not yet been sampled for frogs (e.g., Almeida et al. 2011). Forests on private properties are also important for preserving amphibian diversity in the area (Ferreira et al. 2016). In addition, private properties may function as forest corridors for dispersing and migrating species. We suggest that a program to stimulate the creation of private-owner reserves and ecotourism activities should be implemented in this region. Finally, we have been developing outreach activities (e.g., Bromeligenous Project) with the local farmers, aiming to minimize the anthropogenic effects on anurans. Nevertheless, there is a strong need for a long-term outreach program in the local schools and in the farmlands to protect these forest areas in the future.

## Appendix I

**Vouchers of examined specimens**

*Adelophryneglandulata* (MBML 9560), *Allobatescapixaba* (MZUSP 53559), *Aplastodiscuscavicola* (MBML 9620), Aplastodiscusaff.eugenioi (MBML 7901), *Aplastodiscusweygoldti* (MBML 9540), *Boanaalbomarginata* (MBML 9610), *Boanaalbopunctata* (MBML 9673), *Boanacrepitans* (MBML 9624), *Boanafaber* (MBML 9576), *Boanapardalis* (MBML 9577), *Boanasemilineata* (MBML 9554), *Bokermannohylacaramaschii* (MBML 9552), *Brachycephalusalipioi* (MNRJ 25405), *Ceratophrysaurita* (MBML 591), *Chiasmocleiscapixaba* (MBML 2644), *Chiasmocleisschubarti* (MBML 9599), *Crossodactylodesbokermanni* (MBML 3984), *Crossodactylodesizecksohni* (MBML 768), Crossodactylusaff.gaudichaudii (MBML 15), *Crossodactylustimbuhy* (MBML 13), *Cycloramphusfuliginosus* (USNM 200441), *Dendrophryniscuscarvalhoi* (MBML 8722), *Dendropsophusberthalutzae* (MBML 8589), *Dendropsophusbipunctatus* (MBML 2446), *Dendropsophusbranneri* (MBML 9611), *Dendropsophusbromeliaceus* (MBML 7712), *Dendropsophusdecipiens* (MBML 9590), *Dendropsophuselegans* (MBML 9543), *Dendropsophusgiesleri* (MBML 8795), *Dendropsophushaddadi* (MBML 8775), *Dendropsophusmicrops* (MNRJ 30445), *Dendropsophusminutus* (MBML 9593), *Dendropsophusruschii* (CFBH 37010), *Dendropsophusseniculus* (MBML 9591), *Euparkerellatridactyla* (MBML 7585), Fritzianaaff.fissilis (MBML 46), *Fritzianatonimi* (MBML 8604), *Gastrothecaalbolineata* (MBML 47), *Gastrothecamegacephala* (MBML 9672), *Haddadusbinotatus* (MBML 9621), Hylodescf.babax (USNM 222553), *Hylodeslateristrigatus* (MBML 9595), *Ischnocnemaabdita* (MBML 1143), *Ischnocnemacolibri* (MBML 10568-10572), Ischnocnemaaff.guentheri (MBML 4534), Ischnocnemacf.nasuta (MBML 4667), *Ischnocnemaoea* (MBML 8705), Ischnocnemaaff.parva sp. 1 (MBML 9550), *Ischnocnemaverrucosa* (MBML 9569), *Itapotihylalangsdorffii* (MBML 8585), *Leptodactyluscupreus* (MBML 6845), *Leptodactylusfuscus* (MBML 6003), Leptodactylusaff.latrans (MBML 2077), Leptodactylusaff.spixi (MBML 2439), *Macrogenioglottusalipioi* (MBML 93), *Myersiellamicrops* (MBML 9561), *Ololygonarduous* (MBML 9657), *Ololygonargyreornata* (MBML 2828), Ololygoncf.flavoguttata (MBML 9649), *Ololygonheyeri* (MBML 8581), *Ololygonkautskyi* (MBML 9594), *Phasmahylaexilis* (MNRJ 4120), *Phrynomedusamarginata* (MNRJ 46881), *Phyllodytesluteolus* (MBML 6785), Phyllodytesaff.luteolus (MBML 9658), *Phyllomedusaburmeisteri* (MBML 9581), *Physalaemuscrombiei* (MBML 9542), *Physalaemuscuvieri* (MBML 9579), *Physalaemusmaculiventris* (MBML 9567), Physalaemuscf.olfersii (MBML 2803), Pipaaff.carvalhoi (MBML 4519), Pithecopusaff.rohdei (MBML 9580), *Proceratophrysboiei* (MBML 142), *Proceratophryslaticeps* (MBML 3905), *Proceratophrysmoehringi* (MBML 6409), *Proceratophryspaviotii* (MBML 9585), *Proceratophrysphyllostomus* (MBML 325), *Proceratophrysschirchi* (MBML 9677), *Rhinellacrucifer* (MBML 9575), *Rhinellagranulosa* (MBML 2573), *Rhinelladiptycha* (MBML 687), *Scinaxalter* (MBML 9612), *Scinaxcuspidatus* (MBML 3594), *Scinaxeurydice* (MBML 1128), *Scinaxfuscovarius* (MBML 7820), *Scinaxhayii* (MBML 4707), Scinaxaff.perereca (MBML 508), Scinaxcf.x-signatus (MBML 4542), *Siphonopsannulatus* (MBML 8586), *Siphonopshardyi* (MBML 8909), Thoropaaff.lutzi (MNRJ 1373), *Thoropamiliaris* (MBML 9571), *Thoropapetropolitana* (MZUSP 27725), *Trachycephalusmesophaeus* (MBML 8793), *Trachycephalusnigromaculatus* (MBML 9213), Vitreoranaaff.eurygnatha (MBML 9678), *Vitreoranauranoscopa* (MBML 3725), *Zachaenuscarvalhoi* (MNRJ 84116).
